# Phenome-wide investigation of dietary and health outcomes associated with bitter taste receptor gene *TAS2R38*

**DOI:** 10.1007/s00394-025-03718-6

**Published:** 2025-06-11

**Authors:** Caroline Brito Nunes, Amanda Wei-Yin Lim, Quimbe Dy, Jue-Sheng Ong, Liang-Dar Hwang

**Affiliations:** 1 Institute for Molecular Bioscience, The University of Queensland, St Lucia, QLD Australia; 2 School of Biomedical Sciences, Faculty of Medicine, The University of Queensland, Brisbane, QLD Australia; 3 QIMR Berghofer Medical Research Institute, Herston, QLD Australia; 4 Faculty of Health, Medicine and Behavioural Sciences, The University of Queensland, Brisbane, QLD Australia

**Keywords:** TAS2R38, Supertaster, Genome-wide association studies, Phenome-wide association studies, UK Biobank, Food intake

## Abstract

**Background:**

Bitter taste receptor gene *TAS2R38* determines our ability to perceive the bitter substances phenylthiocarbamide (PTC) and propylthiouracil (PROP). Despite being extensively investigated, its potential correlations with dietary and health outcomes remain inconclusive. This study aims to validate previous findings observed in small studies and explore novel associations using publicly available summary results statistics from large-scale genome-wide association studies (GWAS).

**Methods:**

We examine the associations between three *TAS2R38* variants (rs713598, rs1726866, and rs10246939) and 139 food liking traits and 29 food intake traits using GWAS data from the UK Biobank (N up to 445,779 individuals of European ancestry). We further search for their associations with health outcomes in published GWASs using three online platforms OpenGWAS, Open Targets, and GWAS Atlas.

**Results:**

The bitter sensitive alleles were associated with reduced preferences for and/or consumption of horseradish, salty food, grapefruit, and alcohol (but only for whiskey, red wine, and spirits), increased preferences for cucumber and melon, and increased consumption of tea (p < Bonferroni-corrected threshold of 0.00128). We identified novel associations between bitter sensitive alleles and impaired renal function, indicated by increased serum creatinine levels (p = 9.80 × 10^–5^), decreased urinary proline betaine levels (p = 3.18 × 10^–3^), and an elevated risk of bipolar disorder (p = 4.01 × 10^–5^).

**Conclusion:**

This study highlights the potential impact of the *TAS2R38* genotype on food preference, consumption, renal function, and bipolar disorder. Further research on genetic links will facilitate the development of targeted interventions and public health strategies to mitigate diet-related health risks.

**Supplementary Information:**

The online version contains supplementary material available at 10.1007/s00394-025-03718-6.

## Introduction

Taste plays a critical role in shaping human food choices and food consumption [1–5]. The ability to perceive different chemical compounds in the mouth and distinguish between taste modalities (i.e., sweetness, bitterness, saltiness, sourness, and umami) depends on distinct receptor cells that trigger neuronal signals to generate specific taste sensations [6]. The bitter taste receptors, also known as TAS2Rs, are specialized receptors commonly present in the oral epithelium and responsible for identifying various bitter compounds [7]. Humans possess approximately 26 functional *TAS2R* genes, with variations in these genes affecting individual differences in bitter taste perception [7–9]. Three single-nucleotide polymorphisms (SNPs; rs713598, rs1726866, and rs10246939) within the *TAS2R38* gene determine a person’s ability to taste the bitter compounds phenylthiocarbamide (PTC) and propylthiouracil (PROP), which account for 50–80% of phenotypic variance in the detection thresholds [10–12] and 35–60% of phenotypic variance in the suprathreshold intensity or bitterness [13–15]. Based on the suprathreshold responses, approximately one-third of the population find these bitter compounds tasteless (referred to as “non-tasters”), whilst others find them extremely bitter (referred to as “tasters” or “supertasters”) [10, 16, 17]. Other factors contributing to the phenotypic variance include the expression of *TAS2R38* [18, 19], the number of fungiform papillae [20], and the genotype of the nearby *TAS2R4* [15].

**Fig. 1:**
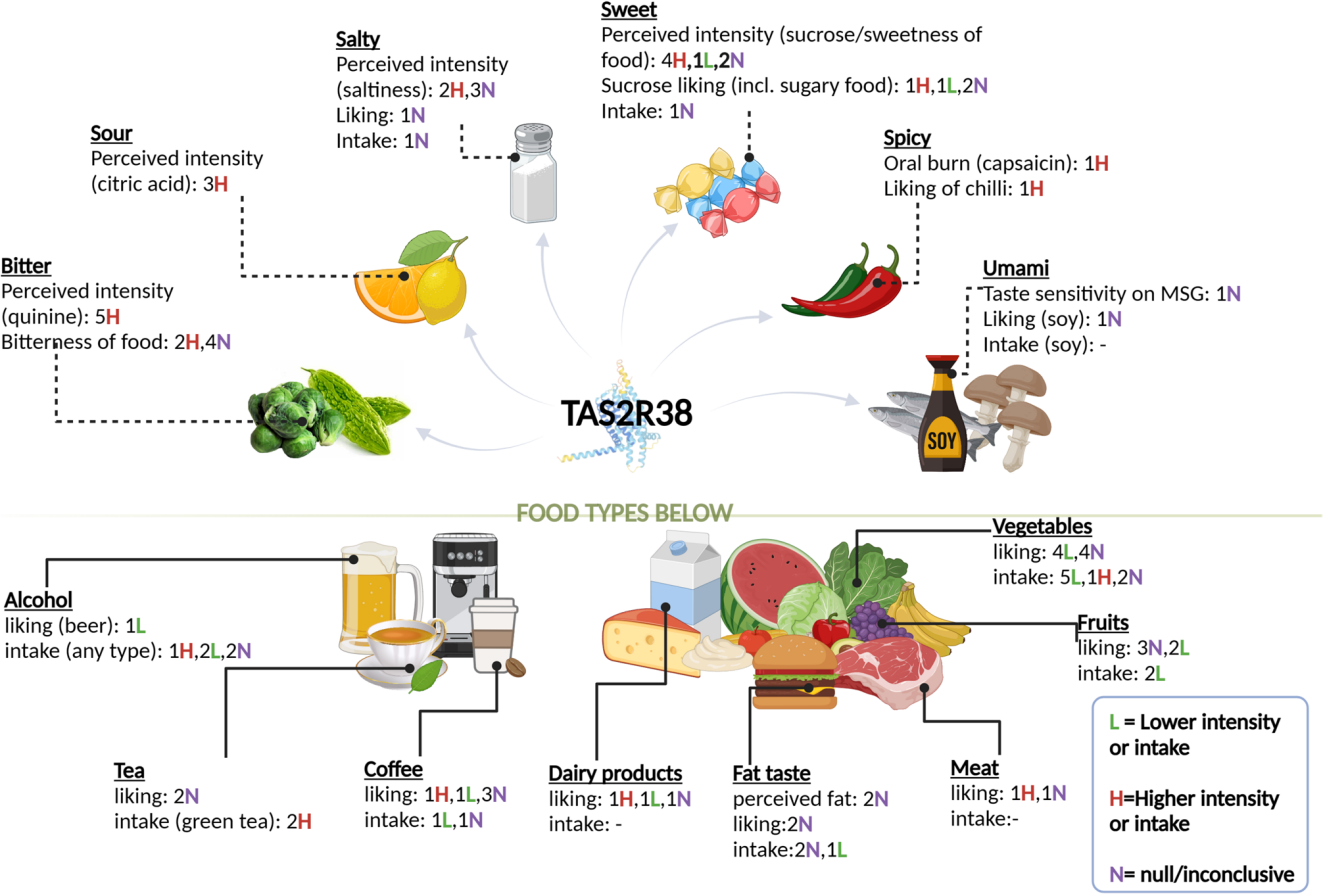
Key findings from the literature on the association between *TAS2R38* genotype and phenylthiocarbamide/propylthiouracil taste perception and dietary behaviour. The direction of association is based on *TAS2R38* bitter sensitive alleles (rs713598 G, rs1726866 G, and rs10246939 C) or PAV haplotype or individuals with increased perceived intensity/bitterness of phenylthiocarbamide or propylthiouracil**.** Numbers indicate the number of studies. Only studies with a sample size > 100 and age > 18 are included in the Figure but the full results are available in Supplementary Table 1

There has been extensive research on the relationship between PTC/PROP taste perception and *TAS2R38* genotype and eating behaviour (Fig. 1; Supplementary Table 1). Apart from a trend showing PTC/PROP tasters or *TAS2R38* tasters (i.e. carriers of *TAS2R38* bitter sensitive alleles) drinking less alcohol and consuming less bitter cruciferous vegetables, many associations remain inconsistent across studies [2, 21–25]. For instance, some studies reported tasters drinking less coffee due to increased perceived bitterness, whilst others found the opposite or no association with coffee consumption [26–33]. Other inconsistent associations include but are not limited to, consumption or liking of a variety of sweet, salty, and fatty foods [3, 4, 4, 34–36]. These inconsistencies could be attributed to differences in the study design, such as variations in phenotypes (e.g., coffee drinker status versus cups of coffee consumed per day among drinkers) and sample characteristics (e.g., sex, age, and ethnicity). Furthermore, several previous studies have utilized small sample sizes, making them underpowered to detect subtle effects accurately. Reporting bias towards significant findings, even without an actual effect, may exacerbate these discrepancies [37]. Therefore, it is necessary to validate these results in larger samples.

**Fig. 2:**
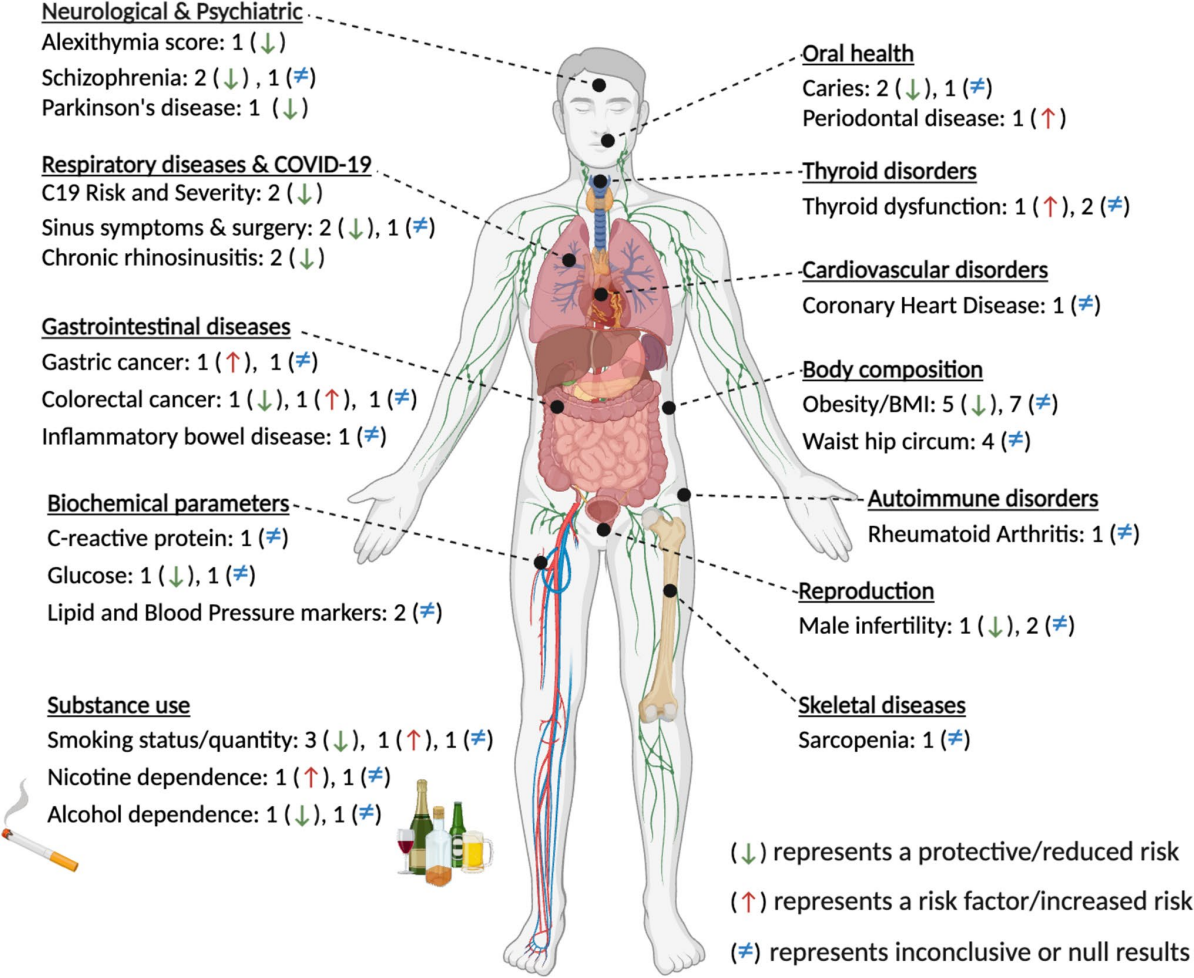
Key findings from the literature on the association between *TAS2R38* genotype and phenylthiocarbamide/propylthiouracil taste perception and health conditions. The direction of association is based on *TAS2R38* bitter sensitive alleles (rs713598 G, rs1726866 G, and rs10246939 C) or PAV haplotype or individuals with increased perceived intensity/bitterness of phenylthiocarbamide or propylthiouracil**.** Numbers indicate the number of studies. Only studies with a sample size > 100 and age > 18 are included in the Figure but the full results are available in Supplementary Table 2

Given that food selection and nutritional status are risk factors for several health conditions, there has also been a great interest in understanding the potential relationship between PTC/PROP taste perception and *TAS2R38* genotype and health outcomes [38] (Fig. 2; Supplementary Table 2). The *TAS2R38* genotype has been linked to the risk of several chronic conditions, such as obesity, cardiometabolic diseases, and colorectal cancer, potentially through its influence on dietary intake [35, 39–42]. TAS2Rs are expressed in extra-oral organs and tissues, including the stomach [43, 44], kidney [45], respiratory tract [46, 47], heart [48], placenta [49] and brain [50], suggesting their potential functions other than taste perception [51, 52]. For example, TAS2R38 receptors play a role in the innate defence of the respiratory system, mainly by increasing mucociliary clearance [46, 53], with different studies showing an association between the *TAS2R38* bitter sensitive alleles and a lower risk of respiratory infections or chronic rhinosinusitis [54–57]. Additionally, both PTC/PROP taste perception and *TAS2R38* genotype have been associated with neurodegenerative diseases, including Parkinson’s disease and schizophrenia, with tasters having a lower risk of these conditions [58–64]. Nevertheless, many findings remain inconsistent across studies, and the mechanisms underlying the association between taster status and health outcomes have yet to be elucidated.

To validate previous findings and explore novel associations, we utilize publicly available summary results statistics from large-scale genome-wide associations studies (GWAS), which examine the associations between millions of genetic variants across the human genomes and traits of interest. Specifically, we investigate the relationship between *TAS2R38* genotype and dietary behaviour using summary results statistics of recent GWASs of food preference and food consumption from the UK Biobank [65, 66], a genetically informative cohort with half a million people from the UK. We further perform a phenome-wide exploratory analysis using three online platforms—OpenGWAS [67], Open Targets [68], and GWAS Atlas [69]—to examine the *TAS2R38* genotype associations across more than a thousand GWASs, covering a broad range of traits, including chronic disease risk and other health-related outcomes.

## Materials and methods

**Fig. 3:**
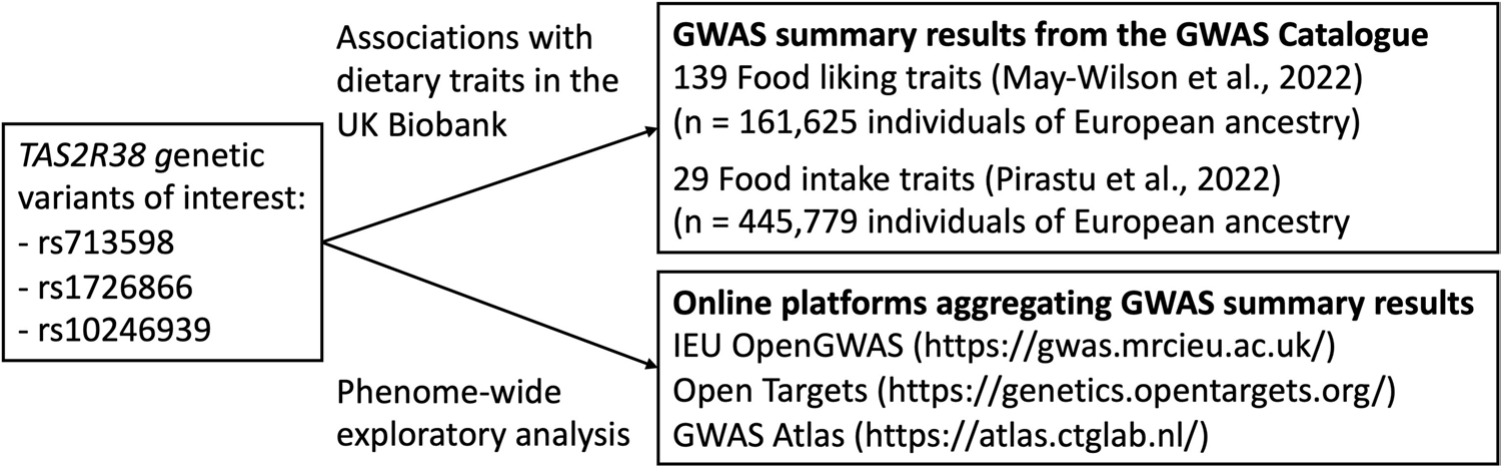
Study design. *TAS2R38* genetic variants were searched for their associations with (i) dietary traits in GWASs of food liking and food intake using data from the UK Biobank, and (ii) all disease and health outcomes in publicly available summary results statistics aggregated in the IEU OpenGWAS, Open Targets and GWAS Atlas

A flow diagram for the study design is shown in Fig. 3.

### TAS2R38 genetic variants of interest

Our analyses were performed using the three *TAS2R38* SNPs that are the primary factor influencing a person’s PTC/PROP taste perception, namely rs713598, rs1726866, and rs10246939, which correspond to the allele changes of C>G, A>G, and T>C, respectively, corresponding to the amino acid changes of A49P, V262 A, and I296 V. Genotypes of these three SNPs are highly correlated (r^2^ > 0.85 in Europeans) and have been commonly investigated as a haplotype difference between AVI (non-taster) versus PAV (taster). rs713598 G, rs1726866 G, and rs10246939 C refer to “bitter sensitive alleles” associated with an increased bitterness perception of PTC/PROP. Here we investigated the associations for each of the three SNPs and reported the effect of the bitter sensitive alleles.

### Food preference and consumption in UK Biobank

We examined the associations between the three *TAS2R38* SNPs in the published GWASs of food liking [65] and food intake [66], both of which used data from the UK Biobank [70]. In brief, the UK Biobank is a population-based prospective study consisting of over 500,000 participants (aged 40–69 years; 54.4% females; 5% of those invited) recruited across 22 assessment centres in the United Kingdom between 2006 and 2010. Participants responded to questionnaires to provide broad information on health and lifestyle in a baseline survey, took part in clinical assessments, and provided biological samples for biomarker and genetic assays.

Dietary intake in UK Biobank was assessed using a touchscreen dietary frequency questionnaire, which included questions about the frequency of consumption of specific foods and beverages over the past year. A total of 29 food consumption traits were included in the GWAS (See Supplementary Tables A and B in Pirastu et al. (2022) for details [66]). Food liking traits were collected through an online questionnaire comprising 152 items, including 139 food and drink items plus additional non-food items that captured liking for health-related behaviours such as physical activity. Participants rated their liking on a 9-point Hedonic scale, with 1 corresponding to “Extremely dislike” and 9 to “Extremely like”. The questionnaire was administered in 2019 to all UK Biobank participants who had agreed to be recontacted by the study. Both studies included only individuals of European ancestry, resulting in up to 445,779 for the food intake GWAS and up to 161,625 participants for the food liking GWAS. Summary results statistics were downloaded from the NHGRI-EBI GWAS Catalog [71] on December 11, 2022, for food liking (GCST90094772; May-Wilson et al. [65]) and food intake (GCST90096926; Pirastu et al. [66]). For the food liking traits, only analyses of rs1726866 and rs10246939 were performed because rs713598 was not available in the GWAS summary statistics.

To account for multiple tests for a large number of correlated traits, the original GWAS of food liking performed eigen decomposition of the genetic correlation matrix and identified 34 independent components that together accounted for > 95% of the overall variance [65]. Similarly, the original GWAS of food intake performed a clustering analysis of all food intake traits and identified 5 main food groups [66]. This study summed the numbers used to account for multiple tests from these studies and set a Bonferroni-corrected significance threshold at p = 0.00128 (0.05/39). Power calculations showed that we had 80% power (alpha level = 0.00128) to detect an association when the *TAS2R38* genotype accounts for > 0.01% of the variance in a food liking trait and > 0.004% of the variance in a food intake trait [72].

### Phenome-wide exploratory analysis

We searched for the three *TAS2R38* SNPs in published GWASs using three online platforms—the OpenGWAS [67], Open Targets [68], and GWAS Atlas [69]. These platforms aggregate human GWAS data, including those from the UK Biobank [70], FinnGen [73], and/or GWAS Catalog [71] summary statistics repository. The three platforms curate summary results statistics from a variety of studies. Our search on disease and health-related traits was performed in February 2023. IEU OpenGWAS only reports associations with a p-value < 0.001, whereas the Open Targets Genetics and GWAS Atlas report those with a p-value < 0.005 and < 0.05, respectively. The effect sizes of SNPs are reported in beta without units and/or odds ratio (OR). The effect sizes are not reported in the GWAS Atlas and are unavailable for some traits in the IEU OpenGWAS. We derived 95% confidence intervals (CIs) for OR using OR and p-value. For associations with missing information (i.e. beta or OR), we downloaded the raw GWAS summary results statistics to extract these data manually. We expected to observe a true association for the same trait in different GWASs or similar traits across GWASs.

## Results

### Food preference and consumption in UK Biobank

**Fig. 4:**
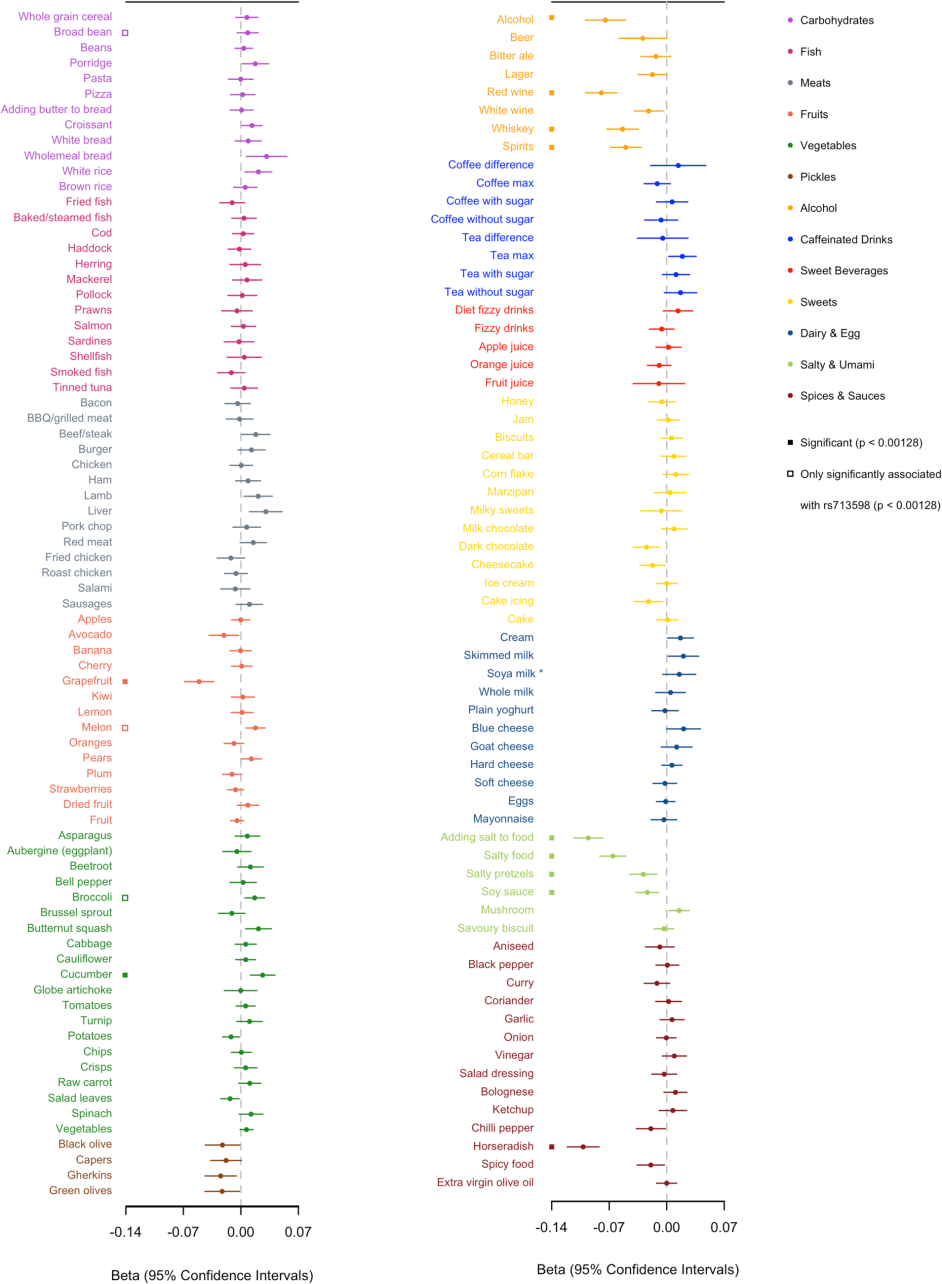
The effect of the bitter sensitive allele rs1726886 G allele on food preference in up to 161,625 participants in the UK Biobank. Solid squares indicate that the association is significant for all three *TAS2R38* variants. Empty squares indicate the association is only significant for rs173598. *Soya milk is categorized with dairy as a milk alternative

**Fig. 5:**
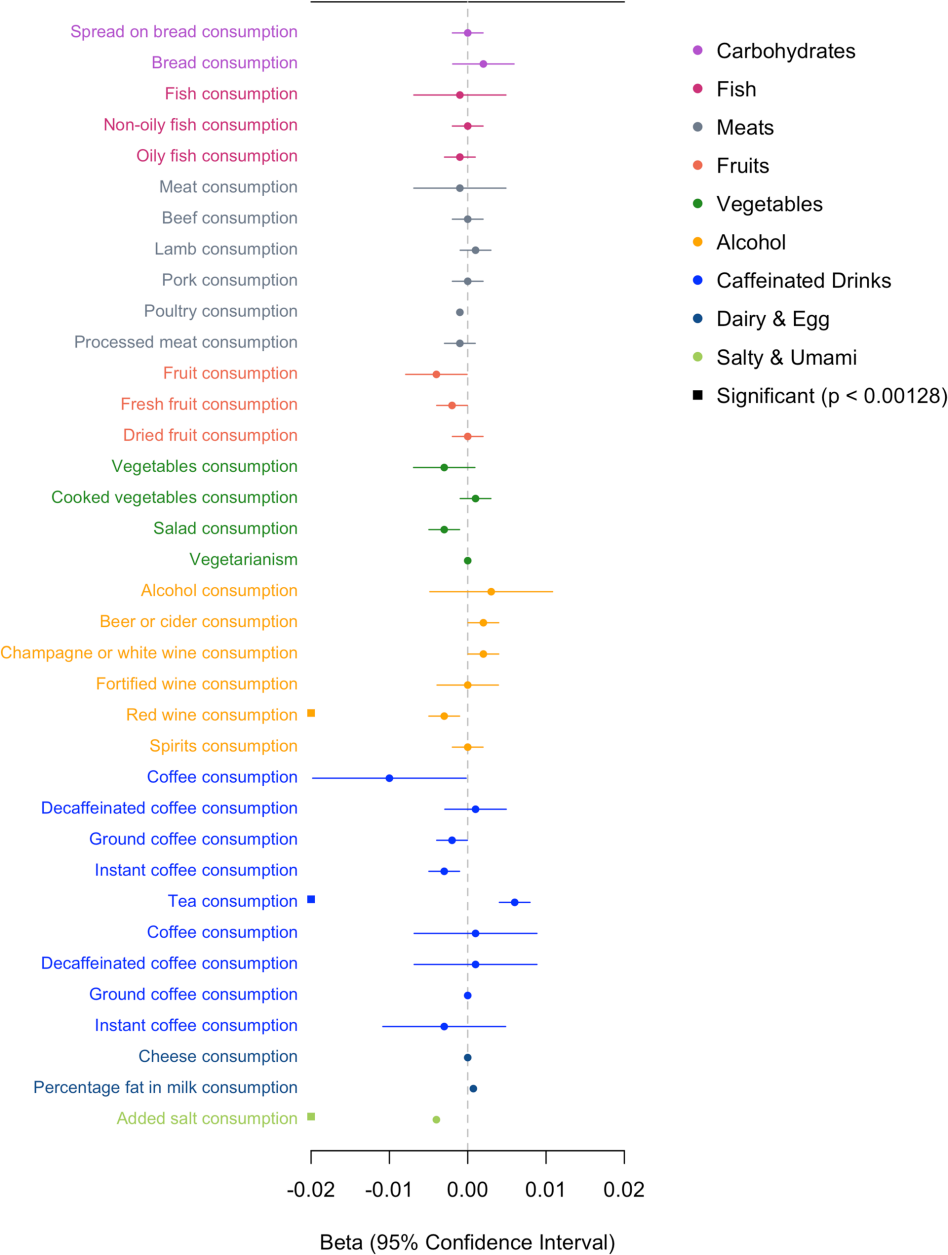
The effect of the bitter sensitive allele rs1726886 G allele on food consumption in up to 445,779 participants in the UK Biobank. Significantly associated traits (solid squares) are the same for both rs1726866 and rs10246939 (rs713598 is not available from the analysis of food intake)

The bitter sensitive allele rs1726866 G was significantly associated with reduced preferences for alcohol (including red wine, whiskey, and spirits), horseradish, grapefruit, added salt, salty pretzels, soy sauce, and salty food, and increased preferences for cucumber (Fig. 4). In the food consumption analyses, the bitter sensitive allele was associated with lower consumption of added salt and red wine but not with other types of alcoholic beverages; the taster allele was associated with higher consumption of tea (Fig. 5). The association patterns for the other two SNPs rs713598 and rs10246939 were very similar, except that the rs713598 G allele was further associated with an increased liking for broad bean, broccoli, and melon (Supplementary Tables 3 and 4). No association was observed for food items in the categories of fish, sweet drinks, and sweets. We did not observe many previously reported associations, including coffee, Brussels sprouts, meat, pickles, and cheese, although some of their subcategories reached the uncorrected significance threshold of p < 0.05. For example, rs1726866 G was associated with lower consumption of salad (p = 0.002) and instant coffee (p = 0.002) but not with total vegetables or total coffee consumption (p > 0.05).

### Phenome-wide exploratory analysis

The bitter sensitive allele rs1726866 G was associated with more than 130 traits with p < 0.05, including 24 traits with p < 0.001 (Table 1; Supplementary Tables 5–7). The strongest association was with a higher risk of bipolar disorder (OR [95% CI] = 1.102 [1.051, 1.155]; p = 4.09 × 10^–5^). The same association with bipolar disorder was observed in another independent study (OR = 1.034 [1.00, 1.070], p = 0.026; Supplementary Table 5); both studies were of European ancestry. Other psychiatric traits associated with the bitter sensitive alleles include increased risk of personality disorder or mental health problems (OR = 1.342 [1.123, 1.603], p = 6.08 × 10^–4^), and lower irritability (beta = − 0.003, p = 3.20 × 10^–4^). Notably, we did not identify any association with Parkinson’s disease, and the association with schizophrenia was only nominally significant in the study that jointly analyzed schizophrenia and bipolar disorder (OR = 1.024 [1.009, 1.039], p = 0.012). Further, we observed a few associations with the volume of brain regions, including the bitter sensitive allele being associated with a larger thalamus (beta = 25.228, p = 1.99 × 10^–4^).

**Table 1:** Associations with P < 0.0001 from the phenome-wide exploratory analysis for the *TAS2R38* rs1726886 G allele using online platforms Open Targets Genetics (OTG), IEU OpenGWAS Project (IOGP), and GWAS Atlas (GA)

Trait	Beta	SE	OR (95% CI)	P	N cases	N overall	Study	Source
Bipolar disorder	0.097	0.024	1.102 (1.051, 1.155)	4.09 × 10^–5^	7647	34,950	Hou et al. [100]	OTG; GA
Creatinine levels	0.008			9.80 × 10^–5^		437,660	Barton et al. [126]	OTG
OTU97_65 (parabacteroides) prevalence	− 0.216	0.055		1.01 × 10^–4^		8956	Ruhlemann MC et al. [101]	IOGP; OTG
Open wounds of head; neck; and trunk	0.089		1.093 (1.042, 1.146)	1.16 × 10^–4^	3837	404,263	UKB SAIGE	OTG
Qualifications: a levels/AS levels or equivalent	− 0.004	0.001		1.50 × 10^–4^		334,070	UKB Neale v1	IOGP
Loud music exposure frequency	0.082	0.022		1.67 × 10^–4^			UKB Pan-Ancestry Broad	IOGP
Anisometropia	− 0.430		0.651 (0.514, 0.824)	1.80 × 10^–4^	155	405,809	UKB SAIGE	OTG
Thalamus volume	25.228	6.780		1.99 × 10^–4^		10,431	Hibar et al. [122]	IOGP
Thyrotoxicosis with toxic multinodular goitre	0.194	0.053	1.214 (1.094, 1.346)	2.21 × 10^–4^			FINNGEN_R5	IOGP
Leukocyte count	0.018			2.27 × 10^–4^		350,470	UKB Neale v2	OTG
Leg pain on walking	0.006	0.002		2.40 × 10^–4^		151,553	UKB MRC-IEU v2	IOGP
Cerebral atherosclerosis	0.350		1.419 (1.166, 1.728)	2.46 × 10^–4^	224	399,241	UKB SAIGE	OTG
OTU99_72 (parabacteroides) prevalence	− 0.203	0.056		2.71 × 10^–4^		8956	Ruhlemann et al. [123]	IOGP; OTG
Irritability	− 0.003	0.001		3.20 × 10^–4^		442,169	UKB MRC-IEU v2	IOGP
Early age-related macular degeneration	0.050	0.014	1.051 (1.023, 1.081)	4.85 × 10^–4^	14,034	105,248	Winkler et al. [124]	IOGP; OTG
Hair colour (natural, before greying): red			0.962 (0.942, 0.983)	5.19 × 10^–4^		385,603	Watanabe et al. [69]	GA
Prospective memory test—time to answer			1.235 (0.535, 1.936)	5.47 × 10^–4^		128,912	Watanabe et al. [69]	GA
CD28 + CD45RA—CD8 + T cell Absolute count	0.088	0.026		5.81 × 10^–4^		3408	Orru et al. [125]	IOGP
Arthropathies	0.024		1.024 (1.01, 1.039)	5.99 × 10^–4^	86,991	260,405	FINNGEN_R6	OTG
A personality disorder|mental health problems ever diagnosed by a professional	0.294		1.342 (1.123, 1.603)	6.08 × 10^–4^	274	117,707	UKB Neale v2	OTG
Pain type(s) experienced in last month: knee pain	0.003	0.001		6.70 × 10^–4^		461,857	UKB MRC-IEU v2	IOGP
Operation code: ectopic pregnancy surgery	0.001	0.000		7.70 × 10^–4^		462,933	UKB MRC-IEU v2	IOGP
Time spent using computer	− 0.006	0.002		8.30 × 10^–4^		360,895	UKB MRC-IEU v2	IOGP
Carbohydrate	− 2.151			8.97 × 10^–4^		51,453	UKB Neale v2	OTG

UKB Neale v1/2, Neale lab analysis of UK Biobank phenotypes, round 1/2; UKB Pan-Ancestry Broad, Pan-ancestry genetic analysis of the UK Biobank performed at the Broad Institute; UKB SAIGE, GWAS of binary traits in the UK Biobank using the software SAIGE; FINNGEN_R5/6, FinnGen biobank analysis round 5/6; UKB MRC-IEU v2, MRC IEU UK Biobank GWAS pipeline version 2. Empty cells indicate that the information is unavailable

*SE* standard error, *OR* odds ratio, *CI* confidence interval, *P* p-value, *N* sample size

The second strongest association was between the bitter sensitive allele and increased serum creatinine levels (beta = 0.008, p = 9.80 × 10^–5^), a biomarker for renal function. Interestingly, the bitter sensitive allele was also associated with many other biomarkers for renal health, including serum non-albumin protein levels (beta = − 0.012, p = 8.95 × 10^–3^), decreased urinary proline betaine levels (beta = − 0.136, p = 3.18 × 10^–3^), and glomerular filtration rate (beta = − 0.008, p = 0.015). Additionally, we identified an association between the bitter sensitive allele and an increased risk of chronic kidney disease (OR = 1.020 [1.006, 1.035], p = 3.08 × 10^–3^), potentially suggestive of a role of TAS2R38 in kidney health and function.

Moreover, we identified several associations with sensory traits. The bitter sensitive allele was associated with increased leg pain on walking (beta = 0.006, p = 2.40 × 10^–4^) and knee pain (beta = 0.003, p = 6.70 × 10^–4^) as well as a higher risk of early age-related macular degeneration (OR = 1.051 [1.023, 1.081], p = 4.85 × 10^–4^), a lower risk of anisometropia, a condition characterized by asymmetrical refraction between both eyes (beta = − 0.43, p = 1.80 × 10^–4^), and a higher exposure frequency to loud music (beta = 0.082, p = 1.67 × 10^–4^).

Although the *TAS2R38* genotype has previously been linked to respiratory tract conditions, we only found weak evidence for an association with respiratory illness or function, such as the risk of hay fever/rhinitis (OR = 1.021 [1.001, 1.041], p = 0.040). However, we did find a trend of the bitter sensitive allele being associated with higher counts of several immune cells, including neutrophil (beta = 0.006, p = 0.004), leukocyte (beta = 0.018, p = 2.27 × 10^–4^) and T lymphocyte counts (beta = 0.008, p = 0.003).

The bitter sensitive allele was associated with a few diet-related traits, including lower daily intake of carbohydrates (beta = − 2.151, p = 8.94 × 10^–4^) and the abundance of *Parabacteroides* in the gut microbiome (OTU97: beta = − 0.216, p = 1.04 × 10^–4^; OTU99: beta = − 0.203, p = 2.71 × 10^–4^). We did not identify any association with body mass index (BMI) or other body composition traits. We observed a few associations between the bitter sensitive allele and cardiovascular traits (cerebral atherosclerosis: beta = 0.350, p = 2.46 × 10^–4^; atrial fibrillation: OR = 1.015 [1.002, 1.09] p = 0.023; hypertension: OR = 1.014 [1.001, 1.027], p = 0.041), but there was no association with the risk of cardiovascular disease. The bitter sensitive allele was also associated with an increased risk of toxic multinodular goiter, a common cause of hyperthyroidism (OR = 1.214 [1.094, 1.346], p = 2.21 × 10^–4^), a decreased risk of oesophagus cancer (OR = 0.858 [0.766, 0.961], p = 4.16 × 10^–3^) and nominally associated with the risk of gastric cancer (p = 1.84 × 10^–2^, unknown direction).

## Discussion

Following a long history of research on *TAS2R38* genotype and PTC/PROP taste perception, this study is the first of its kind to utilize GWAS summary results statistics to investigate the effect of *TAS2R38* genotype on a wide range of phenotypes. This approach is highly cost-efficient as it allows for examining the *TAS2R38* associations in hundreds of thousands of individuals without performing sensory tests or collecting individual genotype and phenotype data. In contrast to traditional lab-based studies that provide more in-depth sensory profiles, GWASs leverage the use of a broader phenotype definition to accrue much larger sample sizes to enable a much larger power to detect small effects. We also discuss the limitations of phenome-wide association analyses and using data from published GWASs.

In the analyses using GWASs of food preference and food consumption among UK Biobank participants of European ancestry, we found that the strongest association was between the bitter sensitive alleles and decreased preferences for adding salt to food. These results support earlier findings showing that PROP tasters found salty food saltier than non-tasters and added less salt to food [3], suggesting a true association among Europeans. Interestingly, while PROP tasters were less likely to salt their food, they tended to consume more salt [3, 74], which could be due to salt’s bitterness-masking and flavor-enhancing effects [3]. The second strongest association detected in this study was between the bitter sensitive alleles and decreased preferences for horseradish, a member of the cruciferous vegetables that contain glucosinolates, a precursor of isothiocyanates whose isothiocyanates (N–C=S) group is responsible for the bitterness of cruciferous vegetables as well as PTC and PROP [75, 76]. Hence, horseradish may carry an extra hint of bitterness, increasing the dislike among tasters. The taste profiles of salt and horseradish may be relatively simple compared to those of other food traits, making them less susceptible to the loss of resolution inherent in food frequency questionnaires and food liking surveys. As expected, we also found a strong association between the bitter sensitive alleles and decreased preferences for grapefruit, which contains a bitter chemical called naringin, which aligns with findings from earlier studies of small sample sizes [77–80]. Surprisingly, we identified a positive association between bitter sensitive alleles and the preference for cucumber, contrasting with previous studies that reported an inverse association in children [1] or limited evidence for an association in a Malaysian cohort [81]. Hence, more work is required to understand the relationship between cucumber preference and *TAS2R38* genotype.

We, however, did not observe the classic association between the *TAS2R38* genotype and preference for Brussels sprouts, a bitter-tasting cruciferous vegetable, due to its high concentration of glucosinolates [82–85]. A possible explanation is that the UK Biobank is an older cohort (median age 58 years old), and taste perception often diminishes with age, potentially due to reduced taste bud numbers and impaired cognitive function [86]. The effect of the *TAS2R38* genotype on PROP perception and vegetable preferences is stronger among children than adults [87]. Furthermore, starting with work by Dutch researcher Hans van Doorn in the 1990 s [88, 89], the food industry has routinely removed bitter compounds like glucosinolates from plant-based foods through selective breeding and other debittering processes [90], possibly affecting results given the UK Biobank’s 2019 food liking survey timing. This could also explain the null association reported in another recent study [21]. Cooking cruciferous vegetables can reduce their bitter taste by denaturing myrosinase, the enzyme responsible for converting glucosinolates to bitter isothiocyanates [91].

As for alcohol drinking behaviour, our results based on the UK Biobank suggest that the effects of the *TAS2R38* genotype vary by the alcoholic beverage. We found associations between bitter sensitive alleles and decreased preferences for red wine, whisky, and spirits, possibly due to these alcohols tasting more bitter and less sweet to PROP tasters than non-tasters [77, 92]. We did not find an association with beer preference in this large cohort, which could explain the inconsistent reports for beer in the literature [77, 93, 94]. As for alcohol consumption, the *TAS2R38* genotype was only associated with red wine consumption but not with spirits consumption. It was reported that increased intake of alcohol was only higher among individuals who were homozygous for the bitter sensitive alleles and similar between those who were heterozygous and homozygous for the bitter non-sensitive alleles [95]. Therefore, a standard GWAS using an additive model rather than a dominant model may lose the power to detect such an effect. It is worth noting that the prevalence of tasters is higher among frequent drinkers compared to the general population, suggesting that while tasters may find alcohol more bitter initially, they may eventually develop a taste for alcoholic beverages, potentially drinking more than frequent drinkers who are non-tasters [21, 96, 97].

We found no association for both preference and consumption of coffee. As the bitterness of coffee can be masked by adding sweeteners and/or milk and other dairy alternatives (e.g., soy milk and almond milk), the relationship between bitter taste perception and coffee drinking behaviour might differ according to the type of coffee (e.g., black coffee vs latte). Interestingly, we did find that bitter sensitive alleles are positively associated with the consumption (but not preference) of another caffeinated beverage—tea. A previous study using data from the UK Biobank showed a negative relationship between the consumption intake of coffee and tea (r = − 0.3) and suggested that PROP tasters are more likely to drink tea than coffee [29]. The same study also reported that the primary taste determinant for the consumption of both coffee and tea is the perception of caffeine rather than the perception of PTC/PROP. This suggests that bitter taste receptors other than TAS2R38 may play a more critical role in determining the drinking behaviour of caffeinated beverages [14]. Furthermore, we note that food flavours are determined by not only bitter taste but also other tastes and smells. Human perception of different foods can be regulated by genetic variations in other sensory receptor genes, such as liking for cilantro, which was associated with a cluster of olfactory receptors on chromosome 11 [98].

While the analyses of food preference and consumption have further reinforced the effects of *TAS2R38* on specific foods and drinks, our phenome-wide exploratory analysis did not find evidence for associations with obesity, BMI or body weight. GWASs of these phenotypes have been performed in extremely large samples, including a GWAS of BMI from 700,000 Europeans [99], and their results are available on the three online platforms. The lack of evidence for an association provides us with confidence that, at the population level, the PROP/PTC taste perception and *TAS2R38* genotype are unlikely to be a predictor for obesity in Europeans. Likewise, we found no strong evidence supporting previously reported associations with cardiovascular disease.

The strongest association in the phenome-wide exploratory analysis was with bipolar disorder, with the bitter sensitive alleles being associated with a 10% increase in the odds of having this condition in a study of 7,647 cases and 27,303 controls [100]. An independent study also identified this association with 20,129 cases and 54,065 controls [101]. While abnormal taste perception can occur during acute episodes of bipolar disorder [102] and changes in the expression of another bitter taste receptor gene *TAS2R5*, 181 kilobases upstream of *TAS2R38*, have been observed in manic episodes in patients with bipolar disorder [103], our study provides the first evidence linking *TAS2R38* genotype to bipolar disorder. Given that *TAS2R38* is also expressed in the brain [50], future investigations are needed to understand the mechanisms underlying the association.

The second strongest association in the exploratory analysis was between the bitter sensitive alleles and higher serum creatinine levels, a biomarker of renal toxicity and poor kidney function [104]. Notably, the bitter sensitive alleles were also nominally associated with chronic kidney diseases and a range of markers of kidney health, including urinary proline betaine levels, glomerular filtration rate, serum non-albumin protein, and glucose levels 2 h after an oral glucose challenge. While the function of bitter taste receptors, including TAS2R38, in human kidney tissue is yet to be elucidated, mouse studies have suggested a potential role of bitter taste receptors in maintaining the structure of renal cells and consequent homeostasis in bodily fluids and electrolytes [49, 105]. Since PROP tasters tend to consume more salts [3, 74], this result warrants further research into how bitter taste receptors regulate kidney function in humans, e.g., through diet or other mechanistic pathways.

While human TAS2R38 receptors are involved in the innate defence system in the upper respiratory system and the bitter sensitive alleles have been previously associated with decreased susceptibility to gram-negative upper respiratory infection and less severe symptoms of chronic rhinosinusitis [46, 53, 55, 56], our exploratory analysis only identified nominal associations with the risk of hay fever and rhinitis and no association with chronic rhinosinusitis. Interestingly, we observed a trend of associations between the bitter sensitive alleles and higher counts of immune cells (i.e., neutrophils, leukocytes, and lymphocytes), which play a crucial part in innate immunity. Together with the recent discovery of *TAS2R38* expression in peripheral immune cells [106–108], our results suggest that TAS2R38 could help control immune responses by regulating immune cell counts.

Finally, we found a novel association between the bitter sensitive alleles and increased prevalence of *Parabacteroides*, gram-negative bacteria in the gut microbiome. Certain species of *Parabacteroides* were associated with human diseases, with a reduced abundance being associated with an increased risk of obesity [109], inflammatory bowel disease [110], metabolic syndrome [111] and many other conditions. *Parabacteroides* also benefit the host by regulating immunity, relieving inflammation, metabolising carbohydrates, secreting short-chain fatty acids, and influencing antibiotic resistance [110–116]. Hence, they play a crucial role in maintaining the host-intestine homeostasis. Our results suggest that TAS2R38 could be involved in regulating the homeostasis of the gastrointestinal tract, possibly through the effect of taste perception on food consumption, which in turn affects gut microbiota. We also found bitter sensitive alleles being associated with other gastrointestinal traits, including oesophagus cancer (with up to 16.5% lower risk) and gastric cancer (unknown direction). Although this contrasts with findings from earlier research suggesting that tasters may avoid bitter cruciferous vegetables and that an unhealthy diet might increase susceptibility to cancer, recent studies propose a diet-independent mechanism, which hypothesizes that the *TAS2R38* genotype may serve as a biomarker for gastrointestinal function and non-tasters may inadequately eliminate harmful chemicals from the gastrointestinal tract [42, 117, 118]. Nevertheless, more research is needed to understand the role of TAS2R38 in gastrointestinal health and function.

This study had a few limitations. First, the analyses on food preferences and consumption were based on data from the UK Biobank participants of European ancestry, so whether our results can be generalized to other populations requires further investigation. The UK Biobank participants were generally older, predominantly female, less socioeconomically deprived, and healthier than the general UK population. These factors need to be considered when comparing our results with other studies [70, 119]. The dietary data were collected using Food Liking and Food Frequency Questionnaires, which are prone to measurement errors due to recall bias, the low resolution of the data (e.g. Food Frequency Questionnaires ask a limited number of foods with limited detail about food preparation), and that the 9-point Hedonic scale with no reference data might not be the most appropriate scale. Further, our exploratory analysis could have been more extensive by the presence of GWAS summary results statistics on the three online platforms and the level of detail provided. For example, the absence of associations for some traits of interest might be due to the p-values failing to reach the reporting thresholds, which vary between platforms. Given that the effect sizes (or direction of association) were not reported for some GWASs or included in the GWAS Atlas [69], the relationships between the bitter sensitive alleles and specific health conditions still need to be clarified. Another challenge in phenome-wide association studies is accounting for multiple testing to reduce type 1 errors [120]. Calculating the degree of false-positive results is not straightforward as there are potential correlations between traits aggregated in the databases. While we are more confident in associations identified in independent studies, they still need to be validated with hypothesis-driven research. In this study, we evaluated the effect of *TAS2R38* genotypes rather than the *TAS2R38* diplotypes. Since the three *TAS2R38* SNPs are highly correlated, the effect of a bitter sensitive allele would correspond to the effect of the *TAS2R38* PAV taster haplotype. Notably, our phenome-wide association results were based on the additivity assumption of genetic effect sizes (consistent with polygenic models and GWAS common variant effect size interpretations) when probing the relationship between specific dietary trait/disease phenotypes against the three highly correlated *TAS2R38* SNPs. While we could not evaluate the effect of rare haplotypes such as AAI, this haplotype has a similar effect as PAV haplotype [17] and is present in less than 3% of the population [121].

## Conclusion

Through a cost-efficient approach using summary results statistics from large-scale GWASs, this study extends current knowledge of the associations of *TAS2R38* genotype and PTC/PROP taste perception for dietary and health outcomes and reveals novel associations, i.e., bipolar disorder, kidney health and gut microbiome, which provide new research directions for understanding the role of this bitter taster receptor gene in human health.

## Supplementary Information

Below is the link to the electronic supplementary material.

Supplementary file1 (XLSX 179 KB)
